# A rare complication following laparoscopic Roux & Y gastric bypass: intussusception—case report

**DOI:** 10.1186/s40064-015-1377-9

**Published:** 2015-10-13

**Authors:** Senol Carilli, Mustafa Arısoy, Aydin Alper

**Affiliations:** General Surgery Department, American Hospital, Guzelbahce Sokak No 20, Nisantasi, 34365 Istanbul, Turkey

**Keywords:** Gastric bypass, Obesity surgery, Intussusception, Bariatric surgery, Complication, Obstruction

## Abstract

Obesity is a growing health problem in most parts of the world. Currently only proven long term effective treatment of obesity is bariatric surgery. Roux & Y gastric bypass together with sleeve gastrectomy are the most employed surgical techniques with acceptable metabolic and surgical complication rates. In this paper we would like to present an unexpected complication of Roux & Y gastric bypass: a retrograde intussusception located in the common limb 17 months after the surgery. As intussusception in adults usually originates from a leading point, there is no such an explanation following Roux & Y gastric bypass.

## Background

Obesity becomes an endemic health problem. Even though dietary, life style modifications and medical treatments offer some modest effects, the surgery still is the only effective long-term treatment. As the overall numbers and the variations of bariatric procedures are consistently increasing, relatively unknown complications are also described.

In this report we are presenting a patient who developed an intussusception following laparoscopic Roux & Y gastric bypass (RYGBP) surgery.

## Case

A 49 years old female patient was admitted to emergency department due to epigastric pain, nausea and non-bilious, non-bloody vomiting lasting 2 days. Her last bowel movement was described as normal in the morning.

Past surgical history included cervical and lumbar herniated disc surgeries, total thyroidectomy due to papillary thyroid cancer 7 years ago and laparoscopic RYGBP 17 months ago in our department. In our department we routinely use linear staplers for the transections and anastomoses as described by Ramos et al. (2014). Following the completion of antecolic gastro-jejunostomy and jejuno-jejunostomy continuous absorbable sutures are used to close Peterson space.

Physical examination revealed slight distention around epigastrium, increased bowel sounds, tenderness with palpation and muscular guarding at left upper quadrant. Her BMI was reduced to 25.4 kg/m^2^ from 39.2 kg/m^2^ following the bariatric surgery.

**Fig. 1:**
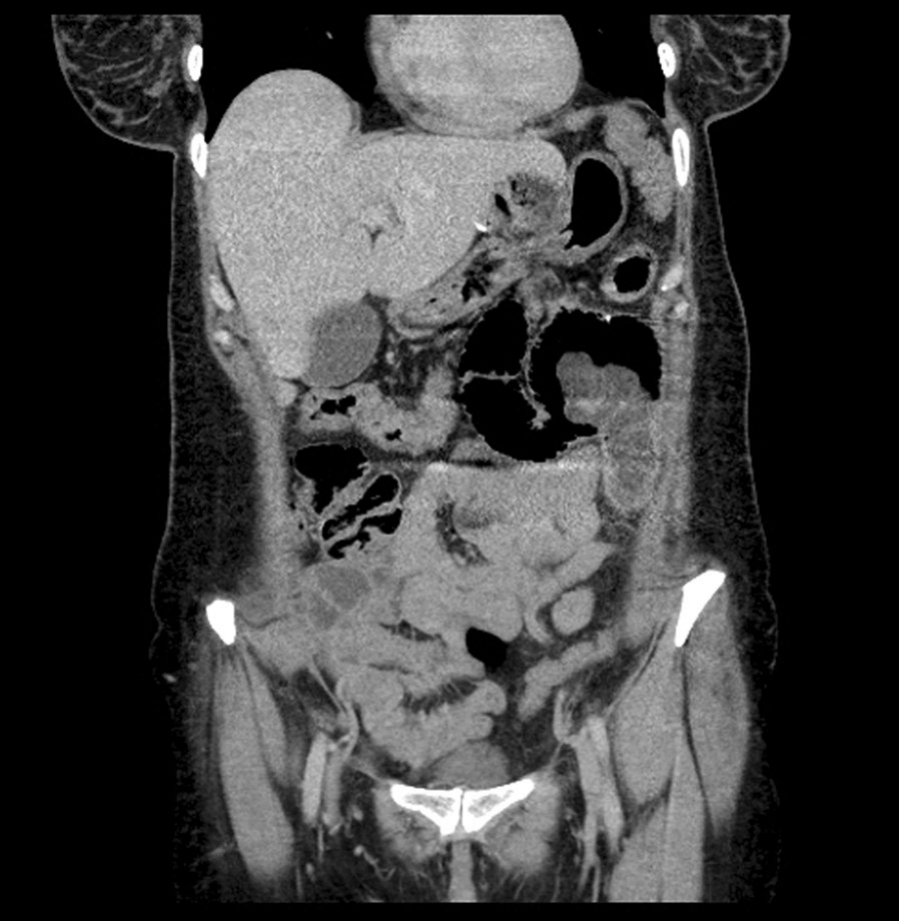
Tube in tube or target sign is characteristic for the diagnosis of intussusception on CT scan

Routine blood tests revealed WBC 11,080/μL (normal 4100–11,000/μL), CRP 17.3 mg/L (normal 0–5). Remaining of the laboratory values were within normal limits. Intravenous contrast enhanced abdominal CT scan showed an intestinal intussusception and small bowel dilatation proximal to it (Fig. 1).

**Fig. 2:**
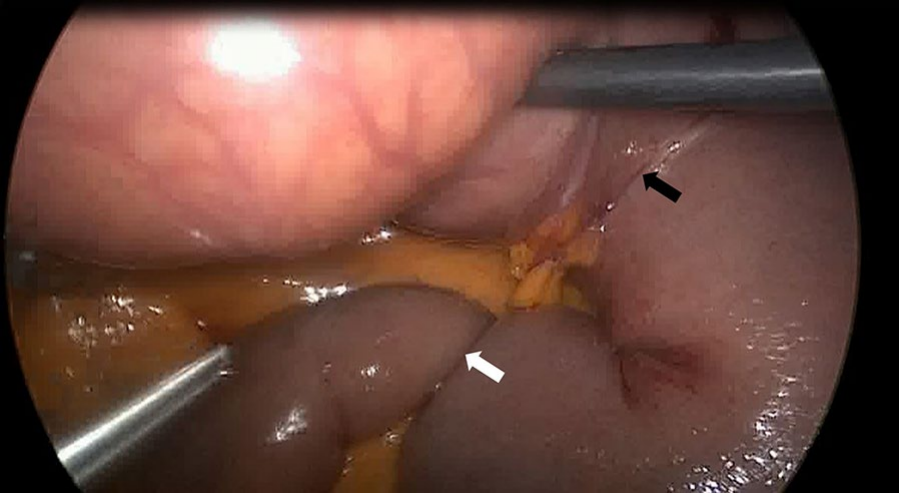
Intussusception was located at 30 cm distal of the jejuno-jejunostomy anastomosis. *White arrow* indicates intussusception, *black arrow* indicates jejuno-jejunostomy anastomosis

An emergent laparoscopic exploration was performed after 3 h of her admission. On exploration, a retrograde intussusception about 30 cm distal to jejuno-jejunostomy was identified (Fig. 2). Length of the invaginated segment was about 20 cm, which was reduced. There was no ischemic appearance but bilio-pancreatic limb was moderately dilated. Per oral feeding was started next morning and she was discharged on postoperative day 3 after an uneventful postoperative course.

She does not have any gastrointestinal complaints since her surgery 9 months ago.

## Discussion

25 % of patients undergoing bariatric procedures require another surgery due to complications or regaining their weight (Singla et al. 2012). Intussusception is relatively rare compared to other well-known complications. It was reported as 0.07–0.6 % (Simper et al. 2010; Duane et al. 2000; Goverman et al. 2004; Wax et al. 2007; Efthimiou et al. 2009). In the medical literature the largest review reported 71 patients and totally less than a hundred case reported till to date according to best of our knowledge (Singla et al. 2012). However, it might be underreported due to its spontaneous reduction potential (Daellenbach and Suter 2011).

Interestingly, majority of the affected patients are female (98.6 %) with the median age is about 35 (Singla et al. 2012). Intussusception can be seen following both open and laparoscopic RYGBP surgeries. It is also reported after loop gastric bypass surgery in which gastrointestinal continuity is less disturbed (Daellenbach and Suter 2011).

Duration of symptoms vary from few hours to few months. In most patients there is no sign of peritonitis and the abdomen is soft, as in our patient.

The classic triad of pain, bloody stools and palpable mass is rarely seen in these cases. Laboratory findings are nonspecific. CT scan is the most valuable diagnostic tool which shows “target sign” or “tube in tube” sign.

Differential diagnoses include internal herniation especially through the Peterson space, bands and volvulus in these patients following laparoscopic RYGBP surgery.

Among the specified cases 86 % have retrograde intussusception (Singla et al. 2012). All three limbs of the Roux & Y anastomosis can be affected by intussusception, but in the vast majority of cases common channel was invaginated through the jejuno-jejunostomy (Daellenbach and Suter 2011).

Mainly there are four options during the surgery; reduction of the intussusception, reduction with plication, resection of the affected bowel and resection of the bowel with revision of the anastomosis.

If there is a bowel ischemia, then the treatment of choice is resection and anastomosis, when the viability or the functions of the segment is questioned.

Even though reduction is a safe approach, resection and reconstruction decreases reoperation rate (Singla et al. 2012; Stephenson et al. 2014). Daellenbach’s review revealed resection with or without reconstruction of the anastomosis yielded 7.7 % recurrence rate while the simple reduction with or without plication yielded 31.5 % (Daellenbach and Suter 2011).

On the other hand, if no ischemic changes in bowel observed, bowel resection or anastomotic reconstruction may not be necessary. Current literature disclosures mostly resections were due to ischemic changes. We think if the bowels are looking non-ischemic during exploration, and the patient has no previous intussusception history, simple reduction might be more suitable.

Its etiology remains obscure. Dysmotility was proposed as most logical explanation. Not surprisingly, disruption of continuity of small intestine and creating anastomoses might change related electro-physiology. New pacemakers should be emerged to provide myoelectric continuity of the small bowel functions. Some authors also suggested the presence of new lead points such as staple lines or sutures and focal nodular hyperplasia as underlying reason (Duane et al. 2000; Wittgrove and Clark 2000; Lessmann et al. 2008). However, hypothesis remain to be proved. Hocking similarly hypothesized that ectopic pacemakers may cause this pathology (Hocking et al. 1991).

“Roux Stasis Syndrome” is also offered as another responsible mechanism for the intussusception (Tu and Kelly 1994). Thinning and elongation of mesenteric fat due to weight loss is thought as another contributing factor for developing intussusception which may cause to lost its cushion effect, increased bowel mobility and instability which are together may increase telescopic movement (Singla et al. 2012; Daellenbach and Suter 2011). Singla et al. suggested etiology of intussusception is multifactorial and both decreasing of mesenteric cushion effect and ectopic pacemakers are contributing simultaneously (Singla et al. 2012).

But all these postulations are far from the explanation of answering the following interesting questions.

- Why most intussusceptions are originated around the same place, i.e. jejuno-jejunostomy anastomosis,
- Why it is much more common in female patients,
- Why it is more commonly retrograde intussusception,
- Why it is not so common among the patients having Roux &Y gastro-jejunostomy for other purposes,
- While the ectopic pacemakers start the activity just after the RYGBP surgery why intussusceptions are delayed up to 3 years,
- While intussusception may develop following loop gastric bypass is it crucial to accused discontinuity of the bowel in etiology?

## Conclusion

Intussusception is a relatively rare complication of RYGBP surgery. Its etiology is still not clearly understood. The most valuable diagnostic tool is CT scan and it is particularly important in early diagnosis. In our case, due to early diagnosis and normal appearance of the intestines after the reduction, we did not perform further resection and anastomosis. However, this may risk recurrences in the future.

## Abbreviations

RYGBP: Roux and Y gastric bypass
CT: computerized tomography
WBC: white blood cell count
CRP: C reactive protein
BMI: body mass index
